# EVOBREATH. Datasets for evolutionary bioenergetics research on anthropology

**DOI:** 10.1016/j.dib.2023.108955

**Published:** 2023-02-06

**Authors:** Ana Mateos, Jesús Rodríguez

**Affiliations:** National Research Center on Human Evolution (CENIEH). Paseo Sierra de Atapuerca 3, 09002 Burgos, Spain

**Keywords:** Human energetics, Metabolic rates, Body composition, Anthropometry, Palaeolithic physical activities

## Abstract

Human bioenergetics has been incorporated into the palaeobiology of human ancestors during the last years to broaden our understanding of Human Evolution. The hypotheses based solely on the taxonomy and phylogenetic relationships of the fossil record, cannot easily explain many of the questions about the physiology of past humans. Data on the energetics and physiology of recent humans, together with comprehensive analyses of body proportions and body composition in relation to human metabolism, are needed to understand the evolutionary constraints of hominin ecophysiology. Furthermore, specific datasets including energetic data from modern humans are required to model hominin palaeophysiology. EVOBREATH Datasets were gradually developed since 2013 to store and manage all the data obtained in the Research Programs on Experimental Energetics developed by the Palaeophisiology and Human Ecology Group and the Palaeoecology of Mammals Group of the National Research centre on Human Evolution (CENIEH, Burgos, Spain). All experimental tests were developed either in the CENIEH BioEnergy and Motion Lab (LabBioEM) or in the field, using mobile devices. Datasets include quantitative experimental data related to human anthropometry (Height, Weight, all postcranial dimensions and segments, including hands and feet, and computation of anatomical indices), body composition (fat mass, fat-free mass, muscular mass, body water), and energetics (resting metabolic rate and energetic expenditure in different physical activities, oxygen consumption (O_2_) and carbon dioxide (CO_2_) production measured breath-by-breath) obtained in multiple studies with in vivo subjects of different ages (adults, adolescents and children) and both sexes (*n* = 501). These datasets are useful to optimize the time-consuming process of generating experimental data and to facilitate their reuse by the scientific community. Researchers can readily employ the datasets in their own research endeavours.

**Specifications Table**

**Table:** 

Subject	Anthropology, Data Science
Specific subject area	Human Energetics, experimental trials on physical activities as walking unloaded, carrying burden, digging, gathering, and stone knapping.
Type of data	Excel files with primary data on energetics during walking unloaded, carrying burden, digging, gathering, and stone knapping. Files with raw graphs and images of indirect calorimetry and ventilatory tests are available upon request. Excel files with primary data on body composition and anthropometry of subjects that participated in the trials.
How the data were acquired	Metabolic rates were measured by breath-by-breath ventilatory indirect calorimetry protocols, which were monitored through oxygen consumption and carbon dioxide production using a Master Screen-CPX JAEGER® device, and analyzed by the LabManager IntelliSupport 5.72 application. All ventilatory trials were performed under standard environmental conditions of temperature, barometric pressure, and relative humidity. Indirect calorimetry devices were calibrated using standard temperature, pressure, and dryness conditions. Locomotion tests were performed on a treadmill (hp COSMOS©). Body Composition protocol was performed using a Bioelectrical impedance vector analysis BIA 101 AKERN® and BodyGram Pro© software (v2010). All anthropometric variables were measured using a Harpenden stadiometer (Holtain Limited) and a Harpenden anthropometer (Holtain Limited) and anthropometric tape. All equipments are located in the BioEnergy and Motion Lab (LabBioEM) at National Research Center on Human Evolution (CENIEH, Burgos, Spain).
Data format	Raw Analyzed Filtered
Description of data collection	Written informed consent to participate and additional informed consent to publish or take photographs were obtained from the all participants in the trials; or, in the case of children and adolescents, from their parents and/or legal guardians. The samples were exhaustively controlled using exclusion criteria that aimed to eliminate certain influencing factors of energetic expenditure, such as medication, food ingestion, or metabolic and cardio-respiratory pathologies.
Data source location	• Institution: National Research Center on Human Evolution (CENIEH). • City/Town/Region: Burgos • Country: Spain • Latitude and longitude (and GPS coordinates) for collected samples data: LabBioEM, 42° 20′ 31.65″ N, 3° 42′ 08.68″ W; Sierra de Atapuerca locations, 42° 20′ 53.72″ N, 3° 31′ 07.61″ W / 42° 21′ 58.53″ N, 3° 32′ 03.97″ W
Data accessibility	Repository name: Mendeley Data Data identification number:
	doi.org/10.17632/rhmdzmx6kk.1 doi.org/10.17632/rvyzmy88c5.1 doi.org/10.17632/jpjn3xt28c.1 Direct URL to data: https://doi.org/10.17632/rhmdzmx6kk.1 https://doi.org/10.17632/rvyzmy88c5.1 https://doi.org/10.17632/jpjn3xt28c.1
Related research article	A. Mateos, G. Zorrilla‐Revilla, J. Rodríguez, Let's Play at Digging: How Vigorous Is This Energetic Task for a Young Forager? Hum. Nat. 33 (2022) 172–195. https://doi.org/10.1007/s12110–022–09428-w

## Value of the Data

• EVOBREATH serve as an efficient tool to improve the knowledge of human physiology in Human Evolution.
• The major strength of EVOBREATH datasets resides in the high quality raw data of up to 500 individuals in different experimental tests.
• The data can be used as part of a reference database for energetics and anthropometry in healthy individuals in disciplines as Anthropology, Biology, Medicine, Sport Sciences, Epidemiology, among others.
• These datasets are useful to optimize the time-consuming process of generating experimental data with in vivo subjects in labs and outdoors and to facilitate their reuse by the scientific community.
• These data are of value to those who are conducting research in human energetics and physiology or any other scientific purpose. Researchers can readily employ them in their own research endeavours.
• The data can be used to investigate associations between energetics, body composition and body dimensions in adults, adolescents and children of both sexes and to compare age and sex in resting metabolism and physical activities.

## 1 Objective

EVOBREATH is a dataset aimed to the analysis of human bioenergetics in an evolutionary perspective. Extensive data on recent human physiology and energetics and on their relationship to body proportions and composition are required to interpret the anatomy and morphology of human ancestors observed in the hominin fossil record. These data are also required to understand the evolutionary ecophysiological constraints of hominin past populations. Some of the raw data recorded in these datasets were analysed in a number of original research articles [1], [2], [3], [4], [5], [6], [7], [8], [9], [10].

## 2 Data Description

**Fig. 1:**
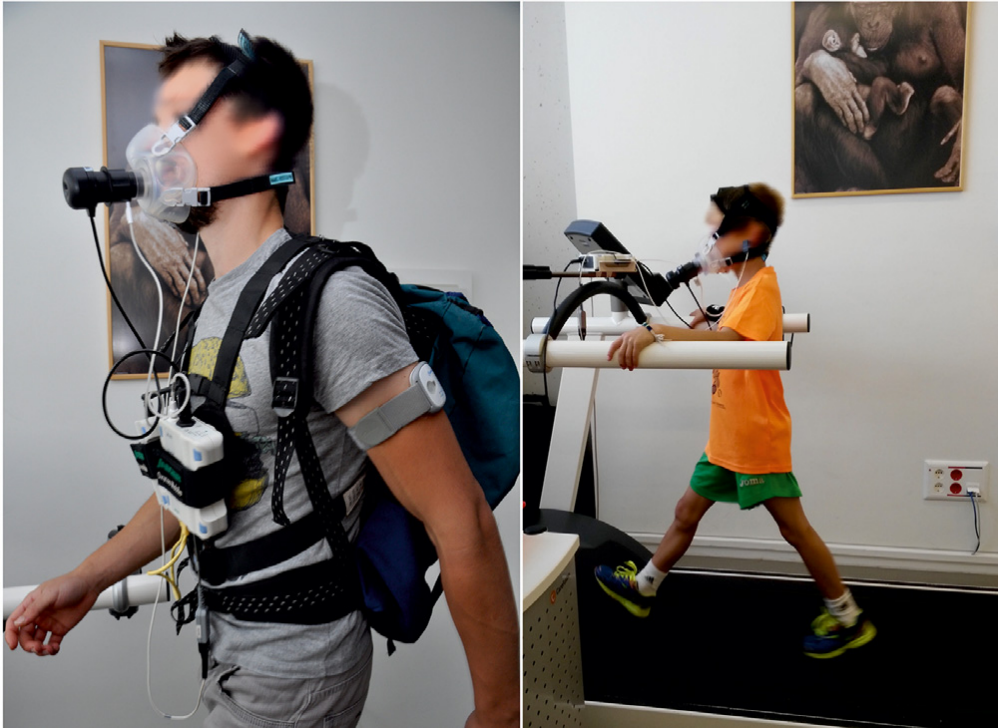
Walking trials in adults and children, performed at LabBioEM in 2015 (Project BioE1- CEIC 1480) and 2017 (Project BioE8-CEIC 1815).

**Fig. 2:**
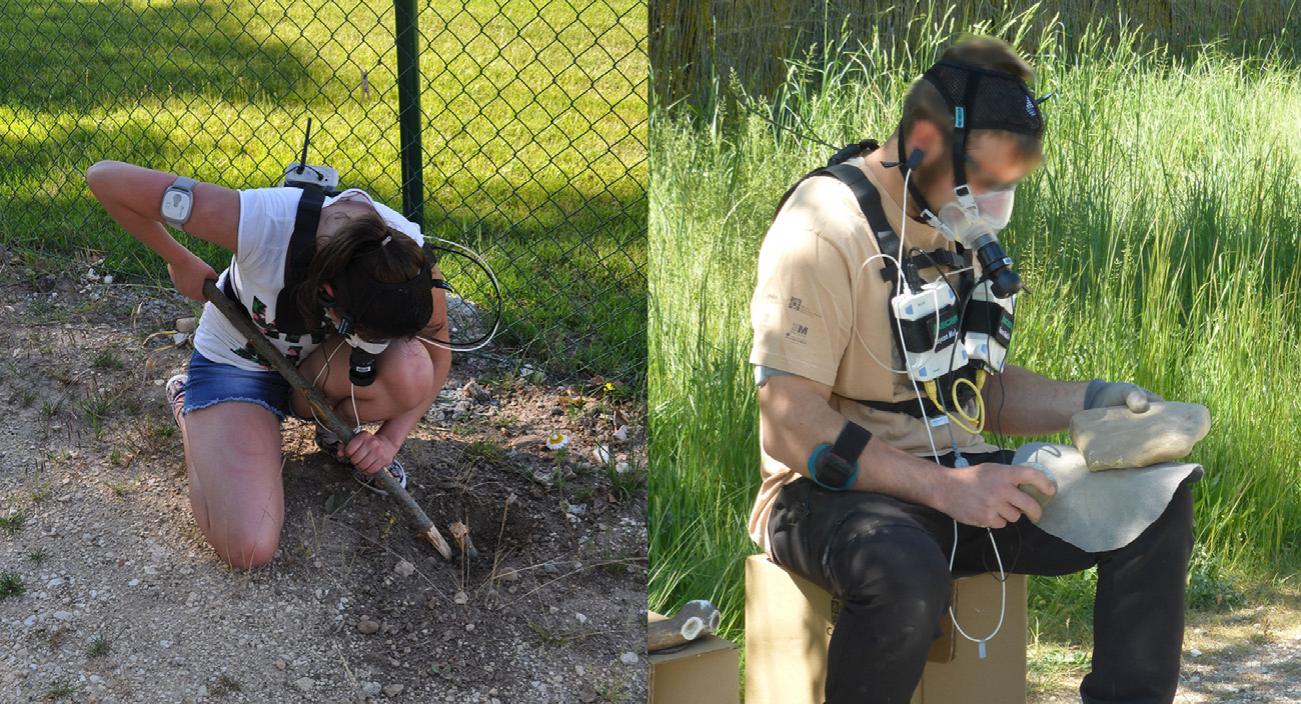
Digging task accomplished by an adolescent and knapping experiment of handaxe production, both performed in 2016 outdoors (Project BioE5-CEIC 1586, Project BioE1- CEIC 1480, respectively).

The raw data shared in the repositories [11], [12], [13] consist on tables presenting information on anthropometric measurements and anatomical dimensions of body segments, proportions of body tissues, and metabolic rates at rest and while performing different physical activities. All data were collected at LabBioEM using different devices (Table 1). The description of all the variables and parameters (Table 2) are included inside the spreadsheets (.xls) and document files (.pdf) of the datasets. Raw graphs of indirect ventilatory test as output produced by LabManager IntelliSupport 5.72 application (JAEGER®) are available upon request. The graphs include the monitored exchange of the O_2_ volume consumed and the CO_2_ volume produced, breath by breath, and the equivalent metabolic rate during the trials (see Figure S1 in [2]). Additionally, some pictures of participants were taken during the tests and informed consent for publication was obtained from volunteers and parents and /or legal guardians. See Fig. 1 and 2 as examples of images illustrating the course of the experimental trials of walking unloaded on the LabBioEM treadmill (Fig. 1) and of digging and stone knapping tasks performed outdoors (Fig. 2).

**Table 1:** Characteristics of the measuring devices used in EVOBREATH datasets. All equipments are located in the BioEnergy and Motion Lab (LabBioEM) at National Research Center on Human Evolution (CENIEH, Burgos, Spain).

Equipment	Description
Master Screen-CPX JAEGER®	Ergospirometer device for indirect calorimetry technique
Oxycon Mobile JAEGER®	Ergospirometer portable device for indirect calorimetry technique
BIA 101 AKERN®	Bioelectrical impedance vector tetrapolar analysis
Harpenden stadiometer	Holtain Stadiometer
Harpender anthropometer	Holtain Anthropometer

**Table 2:** Variables and parameters included in the Mendeley datasets.

Variable	Description	Dataset References
ID	Identification code of the subject	[11,^12^,13]
Sex	M (Male) or F (Female)	[11,^12^,13]
Age	Age of the subject	[11,^12^,13]
Height	Stature	[11,^12^,13]
Weight	Body mass	[11,^12^,13]
FL	Femur length	[11,13]
TL	Tibia length	[13]
BIL	Bi-iliac breadth	[11,13]
BiBr	Bitrochanteric breadth	[13]
UpLL	Upper Limb Length	[13]
Brachial Index	(Radius Length*100) / Humerus Length	[13]
FM	Fat Mass	[11,^12^,13]
FFM	Fat-Free Mass	[11,^12^,13]
RMR	Resting Metabolic Rate	[11,^12^,13]
EE_WW00	Energy expenditure Walking unloaden	[11]
EE_WW05	Energy expenditure Walking carrying 5kg	[11]
EE_WW10	Energy expenditure Walking carrying 10kg	[11]
EE_WW15	Energy expenditure Walking carrying 15kg	[11]
EE_DIG_Net_	Energy expenditure during the digging activity	[12]
EE_Walking_Gross_	Energy expenditure during walking trials at different speeds	[13]

## 3 Experimental Design, Materials and Methods

EVOBREATH Datasets store and manage all the data collected in the Research Programs on Experimental Energetics developed at LabBioEM (Burgos, Spain) since 2013. Volunteers were recruited through advertisements in local media, CENIEH social networks and LabBioEM mail distribution lists. All participants, children, adolescents and adults were urban residents of Burgos. Prior to data acquisition, written informed consent was obtained from the participants and, in the case of children and adolescents, their legal guardians. The samples were exhaustively controlled using exclusion criteria that aimed to eliminate certain factors influencing metabolic cost, such as medication, food ingestion, or metabolic and cardio-respiratory pathologies. Participants were asked to follow the instructions below:

- Do not smoke and do not consume alcohol during the day before testing.
- To be fasting.
- Do not take liquids for 4 h before the test.
- Do not perform physical activity for 4 h before the test.
- Urinate 30 min before the test.
- Do not wear metallic objects.

The experimental tests were carried out in different sessions, indoors (resting metabolic rate and locomotion trials on the treadmill), and outdoors (stone knapping, digging, and gathering trials). At LabBioEM, Resting Metabolic Rate (RMR) was measured via indirect calorimetry while participants lay down for 30 min in supine position on a stretcher while wearing a breathing mask and a heart rate monitor [14]. The milliliters (ml) of O_2_ and CO_2_ inhaled and exhaled, and the equivalent metabolic rate in kilocalories (kcal) [15] were recorded. For outdoor trials (stone handaxe knapping, digging activity simulated tuber-gathering task, and acorn gathering) the energy expenditure was monitored using an Oxycon Mobile JAEGER® portable device. This calorimetry device has been lab-validated as criterion standard system [16] and used in field experiments in re-enacted palaeolithic activities [1,^2^,^4^,^7^,10]. All of the ventilatory tests, both indoors and outdoors, were performed under standard environmental conditions of temperature, barometric pressure, and relative humidity, calibrated automatically using ambient STPD conditions (standard temperature, pressure, and dryness). In a second step, for an exact determination of the lung volume, the measuring system of the JAEGER® devices (Triple V) was to be calibrated (Volume calibration) and finally, the gas analyzers (O_2_/CO_2_) integrated in the device were calibrated by means of gas cylinders containing 5% CO_2_ and 16% O_2_. Metabolic rates were measured by breath-by-breath ventilatory indirect calorimetry protocols, which were monitored through oxygen consumption and carbon dioxide production using a Master Screen-CPX JAEGER® device, and analyzed by the LabManager IntelliSupport 5.72 application. Locomotion tests were performed on a treadmill (hp COSMOS©) (Fig. 1). During a set of locomotion and burden transport trials (BioE1- CEIC 1480), adult participants (*n* = 48, 21 females, 27 males) walked on a treadmill in four different conditions: unloaded, carrying 5 kg, 10 kg, and 15 kg backpacks. Speed was set at 4 km/h, and each trial lasted 10 min. Participants were allowed to rest for 5 min between trials to avoid accumulated fatigue [5,11].

Monitored locomotion trials were carried out in a sample of 74 urban children and adolescents of both sexes, aged 7–14 years, to estimate their optimal locomotion speed, their minimum cost of transport, and their energetic cost of walking (BioE8-CEIC 1815) [3,13]. Thus, participants walked on the treadmill during six phases corresponding to six different speeds (2, 3, 4, 5, 6 and, 7 km/h), with each phase lasting 5 min.

Other trials were aimed to re-enact daily hunter-gatherer tasks (Fig. 2). An extractive foraging activity was simulated with a sample of 40 children and adolescents of both sexes, aged 8–14 years (BioE5-CEIC 1586) [2,12]. The activity consisted on digging out wooden stakes which simulated underground food resources, with the help of a wooden stick, over a 15-minute period. Furthermore, a stone knapping activity was monitored in an experiment with 9 male knappers, aged between 25 and 46 years (BioE1- CEIC 1480) [1]. Participants produced a handaxe from a completely cortical quartzite nodule through direct hard-hammer percussion and direct soft-hammer percussion with a deer antler during 12 min. All the data collected in these experiments are included in EVOBREATH.

The Body Composition protocol was performed using a Bioelectrical impedance tetrapolar vector analysis BIA 101 AKERN® and BodyGram Pro© software (v2010). A standardized technique to perform the analysis of BIA [17,18], according to the requirements established in the Consensus Conference of the National Institutes of Health [19]. Participants were lied in supine position on a non-conductive surface with arms separated from trunk and legs. Thus, an alternating electric current of 50 KHz got into the body through the wrist electrodes and was picked up by two electrodes located on the right ankle, as a sensor, measuring values ​​of resistance (Rz) and reactance (Xc).

Anthropometric measurements of each individual were taken using a Harpenden stadiometer (Holtain Limited) and a Harpenden anthropometer (Holtain Limited) and anthropometric tape. Participants were in a comfortable standing position and they were asked to look straight ahead with shoulders in the normal position. The variables height (cm) without shoes was taken by a Harpender stadiometer and weight (kg) in light cloths was measured using a weighing machine. Body circumferences (to the nearest 0.1 cm) were measured using a non- stretchable anthropometric tape without squeezing the skin. Body segments were measured by a Harpender anthropometer [20].

## Ethics Statements

The experimental studies were approved by the Hospital Universitario de Burgos Ethical Committee (Burgos, Spain) (BioE1- CEIC 1480; BioE5- CEIC 1586; BioE8- CEIC 1815), and complies with the 1964 Helsinki Declaration and its later amendments or comparable ethical standards. Written informed consent to participate and additional informed consent to publish or take photographs were obtained from all the participants in the trials; or, in the case of minors from their parents and/or legal guardians.

## CRediT authorship contribution statement

**Ana Mateos:** Conceptualization, Methodology, Formal analysis, Writing – review & editing, Writing – original draft, Investigation, Supervision, Validation, Data curation, Resources. **Jesús Rodríguez:** Conceptualization, Methodology, Formal analysis, Writing – review & editing, Software, Visualization, Investigation, Validation, Data curation.

## Declaration of Competing Interest

The authors declare that they have no known competing financial interests or personal relationships that could have appeared to influence the work reported in this paper.

## Data Availability

Energy expenditure in children and adolescents during digging activities. Dataset from EVOBREATH 2016-2017 (Original data) (Mendeley Data). Energetic expenditure during walking and load-carriage in humans. Dataset from EVOBREATH 2015 (Original data) (Mendeley Data). Energy expenditure of walking in children and adolescents. Dataset from EVOBREATH 2017-2018 (Original data) (Mendeley Data).
